# Early Detection of Epidemic GII-4 Norovirus Strains in UK and Malawi: Role of Surveillance of Sporadic Acute Gastroenteritis in Anticipating Global Epidemics

**DOI:** 10.1371/journal.pone.0146972

**Published:** 2016-04-26

**Authors:** David J. Allen, Eamonn Trainor, Anna Callaghan, Sarah J. O’Brien, Nigel A. Cunliffe, Miren Iturriza-Gómara

**Affiliations:** 1 Virus Reference Department, Public Health England, London, United Kingdom; 2 Department of Clinical Infection, Microbiology & Immunology, Institute of Infection and Global Health, University of Liverpool, Liverpool, United Kingdom; 3 Pathogen Molecular Biology Unit, London School of Hygiene & Tropical Medicine, London, United Kingdom; 4 Department of Epidemiology & Public Health, Institute of Infection and Global Health, University of Liverpool, Liverpool, United Kingdom; 5 NIHR Health Protection Research Unit in Gastrointestinal Infections, University of Liverpool, United Kingdom; 6 St Helens and Knowsley Teaching Hospitals NHS Trust, Liverpool, United Kingdom; Second University of Naples, ITALY

## Abstract

Noroviruses are endemic in the human population, and are recognised as a leading cause of acute gastroenteritis worldwide. Although they are a highly diverse group of viruses, genogroup-II genotype-4 (GII-4) noroviruses are the most frequently identified strains worldwide. The predominance of GII-4 norovirus strains is driven by the periodic emergence of antigenic variants capable of evading herd protection. The global molecular epidemiology of emerging GII-4 strains is largely based on data from outbreak surveillance programmes, but the epidemiology of GII-4 strains among sporadic or community cases is far less well studied. To understand the distribution of GII-4 norovirus strains associated with gastroenteritis in the wider population, we characterised the GII-4 norovirus strains detected during studies of sporadic cases of infectious gastroenteritis collected in the UK and Malawi between 1993 and 2009. Our data shows that GII-4 norovirus strains that have emerged as strains of global epidemic importance have circulated in the community up to 18 years before their recognition as pandemic strains associated with increases in outbreaks. These data may suggest that more comprehensive surveillance programmes that incorporate strains associated with sporadic cases may provide a way for early detection of emerging strains with pandemic potential. This may be of particular relevance as vaccines become available.

## Introduction

Noroviruses are endemic throughout the human population, and are recognised as the leading cause of acute gastroenteritis worldwide [1, 2]. Norovirus infection presents with sudden onset vomiting and/or diarrhoea 12–24 hours after exposure, although disease is typically self-limiting. Norovirus infection is associated with three main epidemiological patterns: (i) outbreaks of gastroenteritis in healthcare settings [3, 4], and other semi-closed settings; (ii) outbreaks of gastroenteritis linked to contaminated food [5–7]; and (iii) with sporadic cases of gastroenteritis in the community [8, 9]. In the UK, there are an estimated three million cases of norovirus gastroenteritis in the community each year [10], and in elderly and immunosuppressed individuals, norovirus gastroenteritis can contribute to excess deaths [11]. Furthermore, the importance of norovirus gastroenteritis in children less than 5 years of age is increasingly recognised following the decline of rotavirus gastroenteritis after the introduction of rotavirus vaccines [12, 13].

The *Norovirus* genus is a genetically diverse group of non-enveloped viruses within the *Caliciviridae* family. The single-stranded RNA genome is ~7.5kb in length, and is organised into three open reading frames (ORF1-ORF3), which encode the non-structural proteins and major and minor structural proteins, respectively. Genetic diversity in the major capsid protein (VP1) is used to classify noroviruses into five genogroups (GI-GV). The majority of strains associated with disease in humans belong to GI and GII, which are further subdivided into multiple genotypes [14, 15]. The most frequently detected norovirus genotype worldwide is genogroup-II genotype-4 (GII-4).

The predominance of GII-4 norovirus strains in the human population is largely due to the genetic plasticity in the virus’ genome, which leads to a high mutation rate, particularly around surface-exposed positions in the capsid thought to be important antigenic sites [16–18]. The evolution of GII-4 norovirus strains leads to the emergence of genetically distinct strains [19] which periodically may also represent antigenic variants capable of evading pre-existing immunity in the population [20]. Emergence of these antigenic variant strains coincides with strain replacement events and increased levels of norovirus activity worldwide, as observed in 2002/3 [21], 2005/6, 2009/10 [19], and most recently in 2012/13 [22, 23].

The global molecular epidemiology of emerging GII-4 strains is largely based on data from outbreak surveillance programmes mainly from high income countries, however, there have been few studies on the emergence of GII-4 strains among sporadic or community cases globally. Re-analysis of samples collected during long-term surveillance studies of rotavirus in children in Malawi detected GII-4 norovirus strains circulating among this population three years prior to the association of similar strains with worldwide epidemics and the recognition of their global emergence [24]. The aim of the work presented here was to confirm that these strains detected in Malawi possessed the sequence characteristic associated with later epidemic strains within the P2 region of the VP1, where epidemic- variant defining epitopes reside. In addition, we wanted to investigate whether the identification of norovirus GII-4 variant strains among sporadic cases of norovirus gastroenteritis several years prior to their emergence as global epidemic strains may be geographically spread and/or whether they may be associated with sporadic infections across the age groups. In order to do this, we included samples and data from previous studies conducted in the UK.

In the UK, there have been three studies of sporadic cases of infectious gastroenteritis in the community, and general practice: (i) the Infectious Intestinal Disease Study in England (IID1) between 1993–1996 [25, 26], (ii) the Second Study of Infectious Intestinal Disease in the Community (IID2) between 2008–2009 [10], and, (iii) The Structured Surveillance of Infectious Intestinal Disease in Pre-School Children in the Community (Nappy Study) between 2007–2008 [27]. To understand the emergence of GII-4 strains outside of outbreak settings, we characterised the GII-4 norovirus strains detected in these studies. Additionally, we characterised further GII-4 norovirus strains associated with sporadic cases of gastroenteritis in children in Malawi collected 1998–2002. Our data indicate that GII-4 norovirus strains of global epidemiological importance may have circulated in the community up to 18 years before they emerged and were recognised worldwide.

## Materials and Methods

### Specimens

This is a retrospective study that included archived material from 294 anonymised GII-4 norovirus-positive faecal samples or cDNA extracted from them (Table 1). Sample and data archives at the Institute of infection and Global Health, University of Liverpool (Malawi study) and The Enteric Virus Unit, PHE (Nappy Study and IID studies) are compliant with the UK human health tissue act and Caldecott principles. Sample collection and definitions have previously been described in details, and all samples had been obtained for the investigation of the aetiology of gastroenteritis. The samples from Malawi were obtained from children <5 years of age hospitalised due to diarrhoea [28]. The Nappy Study samples were from children with gastroenteritis attending general practice in England and Wales [27]. The Infectious Intestinal Disease (IID) study I samples were obtained from cases of gastroenteritis of all ages in the community or attending general practice and healthy controls in the community [26], whilst IID2 samples were from cases only, in the community or attending general practice, in the UK [10, 29].

**Table 1:** Origin of norovirus positive samples analysed in this study.

	Infectious Intestinal Disease Study in England	Malawi Study	Structured Surveillance of Infectious Intestinal Disease in Pre-School Children in the Community	Second Study of Infectious Intestinal Disease in the Community
**Study Name**	IID1	Malawi Study	Nappy Study	IID2
	**●**	**◆**	**▲**	**■**
**Sampling years**	1993–1996	1998–2002	2007–2008	2008–2009
**Countries**	England	Malawi	England	UK
**Number of samples included**	87	3	19	185
**Reference**	[25]	[24]	[27]	[10]

Approval for use of the IID archive material was granted by the IID Study Scientific Steering Committee. The authors did not have access to any identifying patient information. Samples from Malawi used in this study were stored at the Institute of infection and Global Health, University of Liverpool (Malawi study) and IID and Nappy Study samples were stored at The Enteric Virus Unit, PHE. Informed consent was obtained from the patients (or their guardians if younger than 18 years of age) to store their samples for research purposes, at the time the samples were collected.

### Nucleic Acid Extraction, Reverse Transcription and Norovirus Testing

For specimens collected in all studies, nucleic acid was obtained from faecal material, converted to randomly-primed cDNA and confirmed as norovirus-positive using methods as previously described: IID1 study [26], IID2 [10, 29], The Nappy Study [27], Malawi study [24].

### Amplification of the GII-4 Norovirus P2 Domain for Strain Characterisation

In order to determine GII-4 strain types, the region of the ORF2 gene encoding the VP1 hypervariable P2 domain was amplified by PCR as previously described [18, 30]. Full-length P2 domain amplicons were sequenced using a dideoxy chain-terminator method.

### Phylogenetic Analysis

Sequence analysis was performed using Bionumerics v6.1 (Applied Maths, Kortijk,Belgium) and MEGA version 5 [22]. Amino acid sequences were deduced from nucleotide. Throughout, amino acid motifs are designated here by standard IUPAC single-letter amino acid code. Reference strains obtained from NCBI GenBank were included for comparison, accession number numbers: AF145896, X86557, AY532115, AJ004864, AY532127, AY587988, AY502023, AB22092, EF126965, AB445395, GU445325, JX459908, DQ078794, EF126963. Sequences described in this study were deposited in Genbank; accession numbers KU312298-KU312306.

## Results

A total of 294 GII-4 norovirus strains were characterised from four studies conducted between 1993–2009 in the UK and Malawi (Table 2).

**Table 2:** Numbers of GII-4 strains analysed and variant assignation according to phylogentic analysis. Strains that do not align chronologically with the GII-4 variant circulating during the study period study period are highlighted (bold, italic & underlined) in the table.

	IID1	Malawi Study	Nappy Study	IID2	Total
Study Period	●	◆	▲	■	
Strain variant	1993–1996	1998–2002	2007–2008	2008–2009	
**Camberwell/1987**	4				**4**
**Lordsdale/1993**	6				**6**
**Grimsby/1995**	43				**43**
**Dresden/1997**	22				**22**
**Bochum/1997**	6				**6**
**Farmington Hills/2002**	***1***				**1**
**Hunter/2004**	***3***		2		**5**
**2002–2004 Cluster**		***3***			**3**
**Yerseke/2006**			9	34	**43**
**Den Haag/2006**			8	99	**107**
**Apeldoorn/2007**				51	**51**
**New Orleans/2009**	***1***			***1***	**2**
**Sydney/2012**	***1***				**1**
**Total**	**87**	**3**	**19**	**185**	**294**

### Molecular Epidemiology of GII-4 Strains Detected in UK and Malawi

Within the IID1 Study specimens, 49% of strains belonged to the Grimsby/1995 genetic cluster (n = 43/87), and 25% (n = 22/87) belonged to the Dresden/1997 cluster (Table 2). Both Grimsby/1995 and Dresden/1997 were the epidemiologically contemporaneous strains during the study period.

Within the Nappy Study, 47% (n = 9/19) of strains belonged to the Yerseke 2006a genetic cluster, and 42% (n = 8/19) belonged to the Den Haag 2006a cluster (Table 2). Both Yerseke 2006a and Den Haag 2006a were among the most commonly detected GII-4 norovirus strains worldwide during the study period.

Within the IID2 Study, 53% (n = 99/185) belonged to the Den Haag 2006a genetic cluster, and 28% (n = 51/185) belonged to the Apeldoorn/2007 cluster (Table 2). Both Den Haag 2006a and Apeldoorn/2007 were among the most commonly detected GII-4 norovirus strains worldwide during the study period.

**Fig 1:**
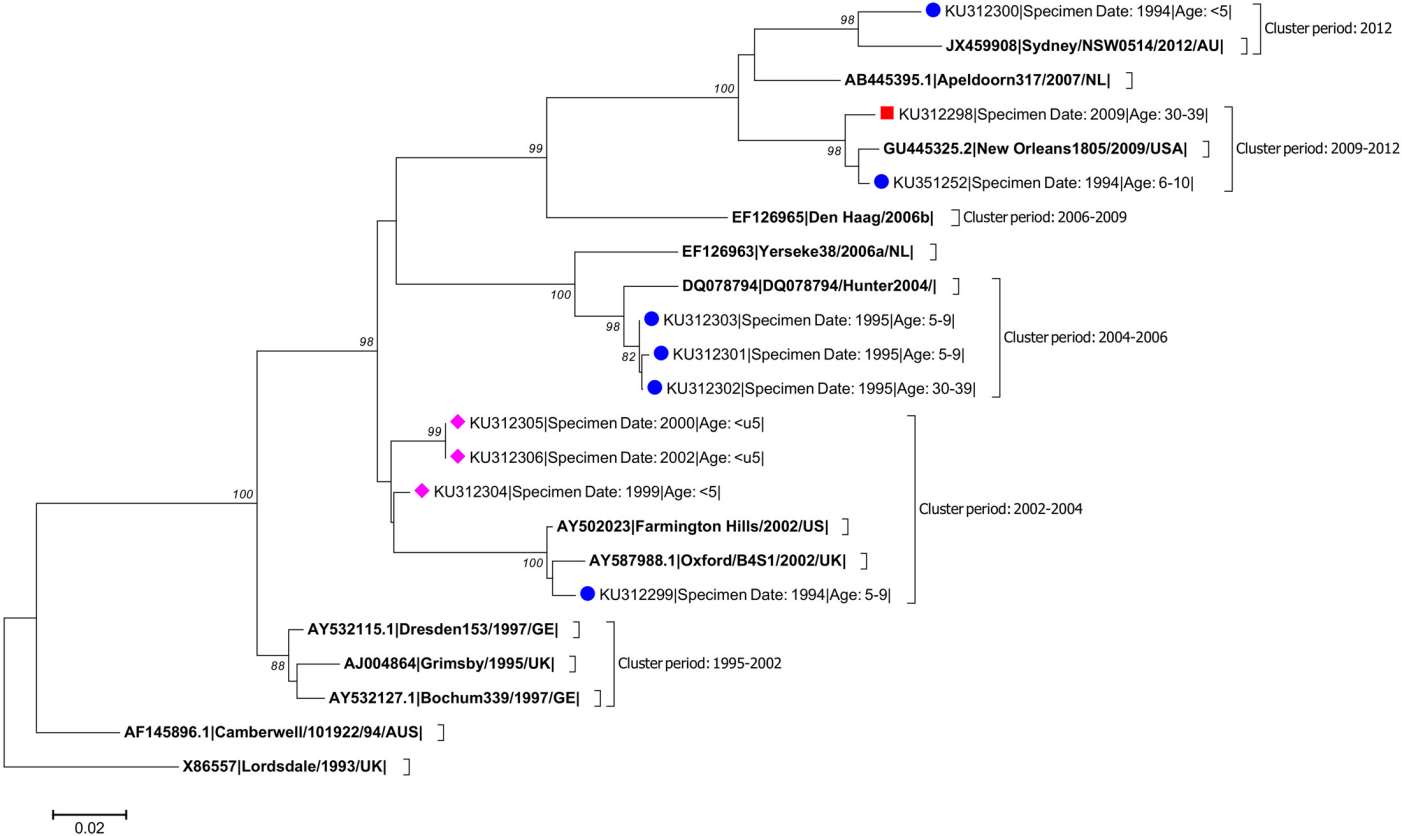
Clustering of norovirus strains identified in sporadic cases of gastroenteritis from Malawi (labelled with pink diamond, as in Table 1), or the UK (labelled with a circle of red square as in Table 1) against global GII-4 strains representative of the epidemic strain variants that emerged between 1995 and 2012. Nucleotide sequences analysed correspond to 470 nt of the P domain of the VP1 encoding gene and encompass the entire length of P2 domain, with the exception of sample EREIID251601, for which only 242 nt were available.

In three of the four studies (IID1, Malawi and IID2), GII-4 norovirus strains were detected in a period that preceded the identification of these strains as global epidemic strains (Table 2 & Fig 1).

Among the IID1 study GII-4 strains, all of which were collected between 1993 and 1996, four strains clustered with strains not identified as globally emerging strains until after 2002. Based on the date of sample collection, and the date these strains were first identified, we retrospectively detected Farmington Hills/2002, Hunter/2004, New Orleans/2009 and Sydney/2012, eight, nine, fifteen and eighteen years prior to their identification as global epidemic strains (Table 2 & Fig 1).

The three strains detected during the Malawi Study cluster together and are related to the GII-4 strains detected after 2002 (Fig 1). Two of the three strains (MW2002 and MW2378) were identified in specimens collected in 1999 and 2000, respectively, two to three years before the Farmington Hills/2002 strain was identified as a global epidemic strain. Phylogenetic analysis indicates that all three strains detected during the Malawi Study are closely related to GII-4 norovirus strains identified in 2002 and 2004 suggesting that strains similar to Farmiongton Hills/2002 were circulating in Malawi at least 2 to 3 years before their identification as global epidemic strains (Table 2 & Fig 1)

Among the four GII-4 norovirus strains detected during the IID2 Study (2008–2009), one example of New Orleans/2009 was detected in a sample collected in the summer of 2009, the period immediately preceding the identification of New Orleans/2009 as a global epidemic strain (Table 2 & Fig 1).

### Analysis of amino acid variation in the hypervariable domain of the major capsid protein

**Fig 2:**
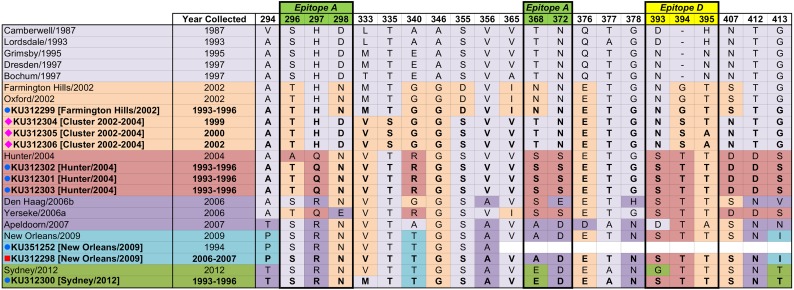
Alignment of the deduced amino acid sequencing obtained from sporadic strains detected in Malawi and the UK with prototype strains representative of the epidemic strain variants that emerged between 1987 and 2012. Successive variants are colour coded and prototype stains are listed chronologically with earliest variants at the top. Study sequences are identified according to origin as in Table 1. Single amino acid identities of the study strains against prototype strains are colour coded using the colour assigned to each epidemic strain variant.

Analysis of variable amino acid positions in the hypervariable P2 domain of these epidemiologically important GII-4 norovirus strains detected in a time period that precedes their identification as global epidemic strains, suggests that these strains represent genetic intermediates between contemporary and emerging GII-4 strains (Fig 2).

The Farmington Hills/2002 strain detected during the IID1 study is identical to the prototype (accession number AY502023). This indicates that the Farmington Hills/2002 strain was in circulation in the UK at least six years prior to its association with global emergence.

The strains isolated from samples collected in Malawi are highly similar to the Farmington Hills/2002 prototype, but show some differences which may suggest they are an intermediated between these and the Grimsby/1995 prototype (accession number AJ004864). In particular, position 298 which is part of Epitope A, known to be important in defining antigenic properties in emerging GII-4 norovirus strains, and position 368, which was shown to be important in the emergence of Sydney/2012. Additionally, positions 355, 365 and 407 are more similar to Grimsby/1995 than Farmington Hills/2002. Two further positions are of note; firstly, position 335 at which a serine residue occurs that is unique compared to all other GII-4 norovirus strains in this analysis. Secondly, position 333 shows more similarity to the later Hunter/2004 prototype (accession number DQ078794) than either Grimsby/1995 or Farmington Hills/2002.

The Hunter/2004 strains detected during the IID1 study are highly similar to the prototype, except at position 296 which shows more similarity to the preceding Farmington Hills/2002 strain. Position 296 is part of Epitope A known to be important in defining antigenic properties in emerging GII-4 norovirus strains.

The New Orleans/2009 strain detected during the IID2 study is identical to the prototype New Orleans/2009 (accession number GU445325). This indicates that the New Orleans/2009 strain was in circulation in the UK at least one year prior to its association with global emergence.

The Sydney/2012 strain detected in the IID1 study is highly similar to the prototype strain (accession number JX459908), except at two significant positions. Firstly, position 333, which is more similar to the Grimsby/1995 prototype (which would have been the predominant GII-4 in circulation during the IID1 study period). Second, position 393, which is part of Epitope D (known to be important in defining antigenic properties in emerging GII-4 norovirus strains), is more similar to the Hunter/2004 prototype.

## Discussion

This retrospective study analyses data from a 25 year period, including GII-4 norovirus strains collected in the UK and Malawi in 1993–2009 from sporadic cases of gastroenteritis, and global epidemic GII-4 norovirus strains identified 1987–2012. Globally recognised most recent epidemic norovirus strains GII- Sydney/2012 and New Orleans/2009 could be detected among sporadic community cases of gastroenteritis up to 18 and 16 years prior to their worldwide recognition as emergent epidemic strains, respectively. Phylogenetic analysis indicated that a small number of sporadic cases of gastroenteritis were associated with norovirus GII-4 strains variants that were not described until several years later. These data also suggest that the diversity among GII-4 strains may be greater among sporadic cases than that seen through outbreak surveillance, and that pandemic strains may circulate at low levels in the community several years prior to their global spread.

GII-4 norovirus strains identified after 2002 display an insertion at amino acid position 394. A small number of strains from the UK and Malawi circulating pre-2002 displayed this insertion. The reasons for this potential delay in their spread are unclear. It is possible that other single amino acid substitutions around this and other proposed epitopes such as those identified in Fig 2 may be key in determining both, strain fitness and immune evasion. These data therefore may be useful in narrowing further the positions alongside the VP1 that may be crucial epitopes for strain-specific immune responses. It is also possible that other regions of the genome, including the non-structural coding genes, may be important for the emergence of pandemic strains, therefore, further characterisation of the whole genomes may provide useful information in this regard. Strains analysed in this study were from cases in children and adults, and no specific age associations were found among the strains that clustered with pandemic stains identified years later.

This work highlights the importance of characterising norovirus strains from sporadic cases in order to gather a more comprehensive picture of the strains circulating in the population and understand the evolutionary processes that culminate in the emergence of pandemic strains. It is possible that the information collected from current norovirus surveillance programmes which are heavily biased towards the characterisation of strains causing outbreaks, represents only strains that evolve from founder variants via a selective sweep following one or a series of bottleneck events in the population. A more systematic approach to strain monitoring which includes norovirus in sporadic gastroenteritis across the entire population may allow the early detection of novel strains and monitoring their progression and establishment in the population. This, coupled with an increase understanding of the epitopes that are important for strain-specific immunity may allow predicting future pandemics. Furthermore, as the prospect of the development and introduction of norovirus vaccines gains momentum, a better understanding of the degree of cross-protection and the role of strain diversity in evading pre-existing immune responses is required. It was recently reported that in a clinical trial in adult volunteers, a consensus a multivalent NoV VLP vaccine induced antibodies that could block carbohydrate ligand binding to GII-4 antigenic variants not yet circulating at the time of vaccination [31]. Our work suggests that epidemic strains circulate in a proportion of the population years before their epidemic explosion, and this could potentially contribute to the observed broad antibody responses to vaccine candidates among pre-exposed individuals.
